# Rapid P300 brain-computer interface communication with a head-mounted display

**DOI:** 10.3389/fnins.2015.00207

**Published:** 2015-06-05

**Authors:** Ivo Käthner, Andrea Kübler, Sebastian Halder

**Affiliations:** ^1^Institute of Psychology, University of WürzburgWürzburg, Germany; ^2^Department of Rehabilitation for Brain Functions, Research Institute of National Rehabilitation Center for Persons with DisabilitiesTokorozawa, Japan

**Keywords:** brain-computer interface, head-mounted display, rapid BCI, P300, locked-in state

## Abstract

Visual ERP (P300) based brain-computer interfaces (BCIs) allow for fast and reliable spelling and are intended as a muscle-independent communication channel for people with severe paralysis. However, they require the presentation of visual stimuli in the field of view of the user. A head-mounted display could allow convenient presentation of visual stimuli in situations, where mounting a conventional monitor might be difficult or not feasible (e.g., at a patient's bedside). To explore if similar accuracies can be achieved with a virtual reality (VR) headset compared to a conventional flat screen monitor, we conducted an experiment with 18 healthy participants. We also evaluated it with a person in the locked-in state (LIS) to verify that usage of the headset is possible for a severely paralyzed person. Healthy participants performed online spelling with three different display methods. In one condition a 5 × 5 letter matrix was presented on a conventional 22 inch TFT monitor. Two configurations of the VR headset were tested. In the first (glasses A), the same 5 × 5 matrix filled the field of view of the user. In the second (glasses B), single letters of the matrix filled the field of view of the user. The participant in the LIS tested the VR headset on three different occasions (glasses A condition only). For healthy participants, average online spelling accuracies were 94% (15.5 bits/min) using three flash sequences for spelling with the monitor and glasses A and 96% (16.2 bits/min) with glasses B. In one session, the participant in the LIS reached an online spelling accuracy of 100% (10 bits/min) using the glasses A condition. We also demonstrated that spelling with one flash sequence is possible with the VR headset for healthy users (mean: 32.1 bits/min, maximum reached by one user: 71.89 bits/min at 100% accuracy). We conclude that the VR headset allows for rapid P300 BCI communication in healthy users and may be a suitable display option for severely paralyzed persons.

## Introduction

Brain-computer interfaces (BCIs) can serve as muscle-independent communication channel through the use of brain signals as control commands for output devices. Event related potentials (ERPs) extracted from the electroencephalogram (EEG) are commonly utilized as control signals (see Kleih et al., 2011; Mak et al., 2011 for reviews). In particular, the paradigm introduced by Farwell and Donchin (1988) is widely applied. It consists of a symbol matrix that is presented to the BCI users on a computer screen. During stimulation, rows and columns of the matrix are highlighted in random order and for spelling the users are asked to focus on the letter or symbol they want to select. The most prominent ERP that is elicited by this method is the P300. It is a positive voltage deflection in the EEG that peaks at about 250–500 ms after onset of a rare, but task relevant stimulus (Polich, 2007). In case of the BCI, the P300 occurs in response to the flashing of the letter the user focusses on. Thereby, the row and the column that contain the target letter elicit ERPs that can be classified and the target letter can be identified by the classifier as the symbol at the intersection of the target row and column. It was demonstrated that most healthy users are able to control a P300 based BCI with accuracies of 80% or higher within the first session (Guger et al., 2009), are able to operate it despite constant distraction and when pursuing the task for a long time (Käthner et al., 2014) and control complex applications such as web browsers (Halder et al., 2015). Severely restricted users (e.g., motor impairments caused by amyotrophic lateral sclerosis, ALS) are also able to gain control over P300 BCIs (Kübler and Birbaumer, 2008; Nijboer et al., 2008; Townsend et al., 2010). However, varying degrees of motor impairments and the different paradigms used yielded a wide range of accuracies (see review by Mak et al., 2011).

Naturally, all visual P300 BCI paradigms require the presentation of stimuli in the field of view of the user. In some situations it is difficult to mount a display such that the user can see all the symbols of the matrix. This holds true especially for a hospital or home environment, where the end user is lying in a bed or sitting in a wheelchair and head movements are restricted due to paralysis. For these situations, we tested whether similar accuracies can be achieved with a virtual reality headset (head-mounted display, HMD) compared to a conventional TFT monitor. First, we tested the virtual reality (VR) headset with healthy users and in a second proof-of-principle step with a person in the locked-in state (LIS) to verify that usage of the VR headset is possible for severely paralyzed persons.

It was shown that similar and satisfactory accuracies (76–88%) can be achieved with a see-through HMD as compared to an LCD monitor (Takano et al., 2011). In their study, participants used a TV control panel with 11 symbols in the matrix and a 2 × 2 light control panel for environmental control. However, speed of selections was relatively slow (15 sequences per selection). Therefore, we aimed at improving speed by using a state of the art stimulation method. Recently, Kaufmann et al. (2011, 2013) showed that P300 BCI performance can be improved by means of face stimuli superimposed on characters of a BCI matrix for flashing, instead of flashing the symbols. Additional ERPs involved in face processing, namely the N170 and the N400f, were elicited as compared to conventional flashing and in some participants, including severely paralyzed end-users, the number of flash sequences needed for correct letter selections could be reduced to one sequence. The N400f is an enhanced negativity that can be observed at parietal and central electrode sites between 300 and 500 ms post stimulus, which is larger for familiar as compared to unfamiliar faces. Thus, it is assumed to be an indicator of face recognition (Eimer, 2000). The earlier occurring N170 component (usually peaking around 170 ms, but latencies up to 240 ms were reported) is most prominent over posterior lateral electrode sites (Bentin et al., 1996; Rossion et al., 1999; Bentin and Deouell, 2000; Joyce and Rossion, 2005; Luo et al., 2010). It is specifically evoked by faces or face components, such as isolated eyes, and not modulated by face familiarity (Bentin et al., 1996; Eimer, 2000). Hence, it is likely that this component represents processes involved in the perception of face specific components (Eimer, 2000). The vertex positive potential (VPP), which was primarily reported in earlier studies as a face specific component, peaks in the same time frame as the N170 over frontal and central electrode sites (Jeffreys, 1989, for a review Jeffreys, 1996). Joyce and Rossion (2005) provided strong evidence that the VPP is another manifestation of the brain process that the N170 represents. In our study, the VR headset, did not allow for the presentation of high resolution images. Since Sagiv and Bentin (2001) demonstrated that even a schematic face can trigger the N170, we overlaid rows and columns of the matrix with stylized representations of a smiling humanoid face (smileys) during stimulation. In a recently published study, Chen et al. (2015) found that similar ERPs were elicited and similar accuracies achieved with smileys as compared to face stimuli applied in a P300 BCI. Thomas et al. (2014) also used smileys as stimuli for a P300 BCI, but did not investigate their specific effects.

For both, spelling with the monitor and the VR headset, a 5 × 5 letter matrix was presented to the study participants. We tested two configurations of the VR headset with healthy participants. In the first, it was configured such that the matrix filled the field of view of the users. This condition is similar to the display on the TFT monitor, where the matrix is also fixed. With this configuration of the headset, we also tested if online spelling with one flash sequence (single trial analysis of EEG) is possible. This configuration could serve persons with severe paralysis as an alternative BCI display method. In the second VR headset configuration, matrix size was increased such that only one letter filled the field of view of the users. The users had to move their heads to focus on individual letters. For this, a built-in 3-axis gyroscope tracks the head orientation of the user. Although potential applications for end users with residual control over head movements could be imagined, we mainly aimed at exploring the possibilities of the headset with this configuration and only tested it with healthy participants; clearly, this approach is not suitable for end-users with severe motor impairment. This condition constitutes a “single stimulus paradigm,” because only the targets are seen (Polich et al., 1994). It is a variation of the classic oddball paradigm that consists of targets and non-targets, the non-targets are omitted in the single stimulus paradigm (Polich et al., 1994). The single stimulus paradigm yielded similar ERP amplitudes compared to the oddball paradigm for auditory (Polich et al., 1994; Mertens and Polich, 1997) and visual stimuli (Wetter et al., 2004).

In our study we aimed at exploring if similar or higher accuracies can be achieved with the single stimulus paradigm. Because single stimuli were substantially larger and thus, more salient as compared to the other conditions and targets were presented in the center of view, we expected P300 amplitudes to be larger.

## Methods

### Participants and data acquisition

Eighteen healthy participants took part in the study (10 female, 25 ± 3.9 years, range: 21–34). They received 8€ per hour as compensation. In addition, one person participated diagnosed with amyotrophic lateral sclerosis (ALS). She was 80 years of age, in the locked-in state (LIS) and communicated with horizontal eye movements. She was artificially ventilated and fed and had an ALS-functional rating scale score of 0 (= worst possible). The study was carried out in accordance with the guidelines of the Declaration of Helsinki and all participants gave informed consent prior to the start of the experiment.

Data was recorded with eight active electrodes positioned at Fz, Cz, P3, P4, PO7, POz, PO8, Oz, and mounted in an elastic fabric cap. The reference electrode was attached to the right earlobe and the ground was positioned at FPz. The data was digitized at 256 Hz and amplified with a g.USBamp amplifier (g.tec GmbH, Austria). A low pass filter of 30 Hz, a high pass filter of 0.1 Hz and a notch filter around 50 Hz were applied. Stimulus presentation and data acquisition were controlled by the BCI2000 software framework (Schalk et al., 2004) with a Hewlett-Packard ProBook 6460b (dual-core CPU, 4 GB of RAM, 64-bit Windows 7). Depending on the experimental condition, stimuli were either presented on an external 22 inch TFT display or with a VR headset (Oculus Development Kit 1, Oculus VR, Inc., USA). The VR headset featured a 7 inch LCD display positioned behind two lenses that allows for a field of view of 90° horizontal. The resolution of the LCD was 1280 × 800 pixel and, thus, approximately 640 × 800 pixel per eye. A combination of 3-axis gyroscope, magnetometers and accelerometers enabled head orientation tracking. The whole headset weighted approximately 380 g.

### Procedure

All healthy participants performed the experimental protocol outlined below, which consisted of online spelling with a BCI using three different display methods. The tasks were the same for all display methods.

We used an altered version of the classic P300 speller paradigm introduced by Farwell and Donchin (1988). A 5 × 5 matrix was displayed consisting of all letters of the alphabet (except Z) instead of a 6 × 6 matrix due to the limited resolution of the VR headset. Kaufmann et al. (2011, 2013) could show that flashing characters with famous faces can improve BCI classification accuracy, because additional face specific ERPs are elicited. Rows and columns of the matrix were overlaid with stylized representations of a smiling humanoid face (smileys) during stimulation. Rows and columns were highlighted in random order. To select a letter, participants were asked to focus on that letter and silently count whenever it was highlighted (overlaid with smileys). Stimulus duration was set to 62.5 ms and the inter-stimulus interval to 125 ms. There was a pause of 10 s between letter selections.

Participants had to perform two screening runs in which they had to spell the words SONNE (engl. “sun”) and BLUME (engl. “flower”). In these runs each row and column was highlighted 10 times. The data of these screening runs was used to obtain the classification weights employing stepwise linear discriminant analysis (SWLDA, see Section Online Signal Classification). These were subsequently used for online spelling. In two runs, they spelled the words LUFTSCHLOSS (engl. “castle in the air”) and SOMMERNACHTSTRAUM (engl. “summer night's dream”). The word to spell was displayed in a line above the matrix with the current letter in parenthesis behind the word to spell. Participants were also instructed verbally about the word and letter to spell. During online spelling each row and column was highlighted three times only. Feedback of the selected letters was presented in a row above the matrix.

**Figure 1:**
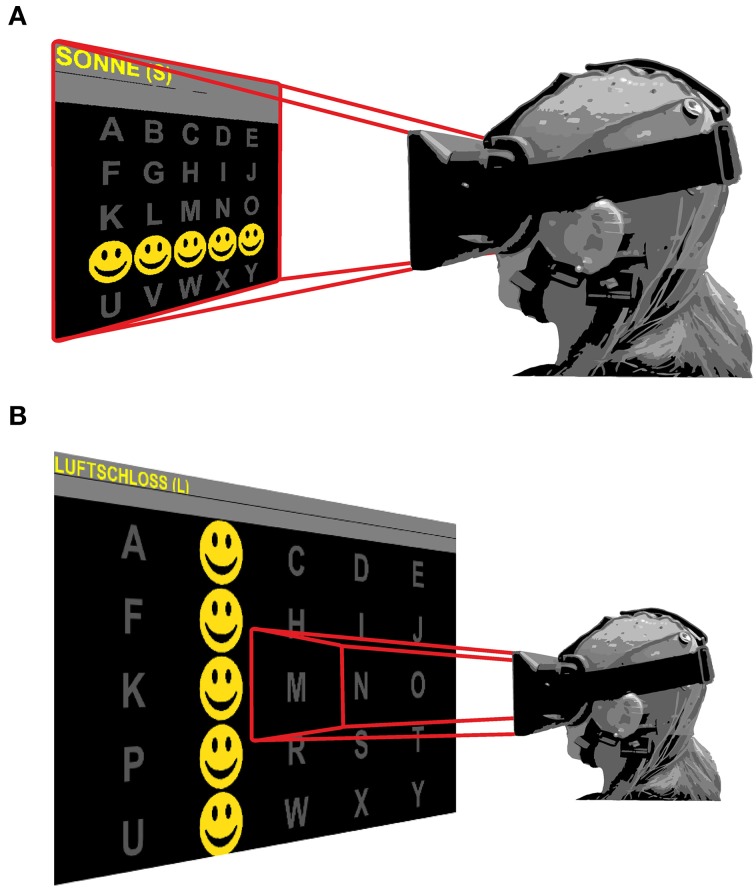
**Field of view for the glasses **(A)** and glasses **(B)** condition**. In **(A)** the display is fixed and the user sees the whole matrix, independent of his/her viewing direction. In **(B)** the user sees only a limited portion of the matrix (one letter) of the matrix and has to move his head to focus on a particular letter. Rows and columns are flashed with yellow smileys during stimulation.

Three experimental conditions (different display methods) were tested. In the first condition, stimuli were presented on a 22 inch TFT monitor. In the second and third conditions, stimuli were presented with the VR headset. The field of view was configured to either show the whole matrix (glasses A condition) or to display only a part of the matrix (glasses B condition; see Figure 1). In the glasses B condition individual letters filled the field of view of the user. Head movements were required to focus on the current letter to spell and/or look at the feedback of the selected letter, which was displayed in a line above the matrix. Additionally, feedback was provided verbally by the investigator in this condition. To control for order effects, the order of the display methods was alternated. To one third of the participants (*n* = 6) the stimuli were first presented on the external monitor, followed by the glasses B (single stimulus) and the glasses A (oddball) condition, while the order for the second group (*n* = 6) was: glasses B, glasses A, monitor and for the third group (*n* = 6): glasses A, monitor, glasses B.

After the glasses A condition, all participants performed an additional online spelling task, copy spelling the word SOMMERNACHTSTRAUM, with a reduced pause between letter selections (2 s) and each row and column was only overlaid once.

Spontaneous remarks of the participants concerning the usability of the display methods were noted.

For the participant in LIS, we adapted the protocol to her abilities. In separate sessions we tried to establish BCI control with three different display methods (a 32 inch LCD flatscreen TV, a 22 inch TFT computer monitor, and the VR headset). The protocol always consisted of screening runs (spelling of words with 5 or 6 characters each) used to obtain classification weights that were applied during subsequent online spelling of 15–29 characters. We either asked her to copy spell (CS) a particular word or she could spell a word of her choice (free spelling, FS). In the latter case, we asked her afterwards which word she had tried to write. She indicated the letters she intended to write in a partner assisted scanning approach with a conventional letter board.

Stimulation parameters were the same as for the healthy participants, with the exception that for the screening runs and online spelling, 10 flash sequences were used. The participant had a limited attention span and indicated via eye movements when she wanted to stop spelling. The participant was naïve to BCI, when we first visited her. Since the glasses B condition required head movements, only the glasses A condition was tested. We performed a total of eight sessions. The different paradigms used in the individual sessions and number of letters for online spelling are listed in Table 1. During all sessions except one, the participant was seated in her wheelchair. In session five, she was in a lying but elevated position in her bed. At the time of our measurements she used the 32 inch flat screen monitor on a daily basis to watch TV. The position of the monitor was kept constant across sessions in front of her wheelchair in her field of view (about 2 m distance to her head). The 22 inch monitor was positioned on a table with a distance of approximately 60 cm to her eyes. The VR headset was attached to her head with the elastic fabric bands provided by the manufacturer. In every session (for all display methods), we made sure that she could see all elements of the matrix and moved/adjusted the displays if necessary.

**Table 1:** **Overview of sessions performed by the participant in LIS**.

**Session**	**Display method**	**Flash image**	**Letters spelled to train classifier**	**Number of letters spelled with feedback**		
					**Spelled with improved classification**	**Total number of letters spelled with feedback**
1^◦^	32″ LCD TV	Einstein face	BRAIN	7 (CS)	5 (CS)	18
					6 (CS)	
2	32″ LCD TV	Einstein face	BRAIN	5 (CS)	10 (FS)	15
3	VR Headset (Glasses A)	Smiley	SONNE	6 (CS)		15
			ERWIN	4 (FS)		
				5 (FS)		
4.1	22″ TFT monitor	Smiley	SONNE	No online spelling		n/a
			ERWIN			
			BLUME			
4.2	VR Headset (Glasses A)	Smiley	SONNE	6 (CS)	10 (FS)	16
			ERWIN			
5^*^	VR Headset (Glasses A)	Smiley	SONNE	6 (CS)		18
			ERWIN	6 (CS)		
				6 (FS)		
6	22″ TFT monitor	Smiley	SONNE	5 (CS)	5 (FS)	24
			BLUME		11 (FS)	
					3 (FS)	
7	22″ TFT monitor	Smiley	SONNE	4 (CS)		29
			BLUME	8 (FS)		
				12 (FS)		
				5 (FS)		
8	22″ TFT monitor	Smiley	SONNE	5 (CS)	6 (FS)	17
			BLUME		6 (FS)	

◦ *16 electrodes used instead of 8*.

* *Problems with artificial ventilation*.

*The category “Number of letters spelled with feedback” is separated into three columns. The rightmost column lists the total number of letters spelled with feedback for each session. The two columns to the left of it provide more detailed information. Each number in these two columns indicates the length of one word that was spelled. Words were either spelled in copy spelling (CS) or free spelling mode (FS). In some cases, letters spelled with feedback were added to train the SWLDA to improve classification for spelling of the subsequent letters. Thus, the number of letters spelled with improved classification, are listed in the middle column*.

A deterioration of the health status of the participant prevented us from conducting further sessions. Damage to the cornea was diagnosed, requiring the eyes to be sealed shut for long periods throughout the day and night and additionally the participant's ability to move the eyes decreased further.

### Online signal classification

A multiple regression algorithm was used for online classification derived by a stepwise linear discriminant analysis (SWLDA). This is an established method that is widely used in BCI studies. In a study comparing SWLDA with other linear and non-linear classification algorithms, the authors concluded that SWLDA provides the best characteristics for practical P300 Speller classification (Krusienski et al., 2006). Details of the analysis are described by Krusienski et al. (2008). The data was segmented into post stimulus epochs of 800 ms and then moving average filtered and decimated, corresponding to a sampling rate of 20 Hz. SWLDA then determined features from voltage values from each of the eight electrodes. For every participant, the features and feature coefficient were determined with Matlab (The MathWorks, version R2012b) using the SWLDA algorithm implemented in the P300-GUI (part of the BCI2000 software). The maximum number of features to be included was set to 60. After the parameters had been determined, based on the data acquired in the screening runs, they were used for online classification during BCI operation.

### Effectiveness and efficiency metrics

The online spelling accuracy was calculated as the number of correctly spelled letters divided by all letters to be spelled. The accuracy was also calculated offline for one, two, and three flash sequences for all experimental conditions.

As a measure of the efficiency of the system the information transfer rate was calculated (Shannon and Weaver, 1964). Firstly, the bitrate was computed with the following formula

$$B={\text{log}}_{2}\;N+P\;{\text{log}}_{2}\;P+(1-P)\;{\text{log}}_{2}\left(\frac{1-P}{N-1}\right).$$

where *N* represents the number of possible targets (25 in case of the 5 × 5 matrix) and *P* is the probability of correct classification (average spelling accuracy). In a second step, the bitrate is multiplied by the average number of selection per minute to obtain the information transfer rate in bits/min.

Further, the correctly selected letters per minute were calculated as a practical performance indicator that can be easily interpreted.

### Offline data analysis

Statistical analysis was performed with SPSS 19. To reveal the effects on spelling performance, we performed a repeated measures ANOVA with the factors “condition” (monitor, glasses A, glasses B) and “number of flash sequences” (one, two, three). To explore if the order of presentation had an influence on spelling performance, we conducted a second repeated measures ANOVA with the factors “order of presentation” (first, second, third) and “number of flash sequences” (one, two, three). For significant main effects, *post-hoc t*-tests were calculated for pairwise comparisons (Bonferroni corrected).

The EEG was analyzed with the EEGLab toolbox (version 10.2.2.4b; Delorme and Makeig, 2004) and the ERPLab plugin (Lopez-Calderon and Luck, 2014) implemented in Matlab (version R2012b). The EEG data was segmented into epochs of 800 ms starting at the onset of a stimulus and baseline corrected with a pre-stimulus interval of 200 ms. Averages were calculated for targets and non-targets. In case of the glasses B condition, flashes were the targets and non-flashes the non-targets. In the periphery of the headset the flashing of the neighboring non-target letters could be noticed. For the healthy participants this procedure resulted in 168 target and 672 non-target trials per condition per participant. Grand average ERP waveforms were calculated for each condition (display method).

We calculated *R*^2^ plots as an estimation of the class discriminative information for specific time frames. On the basis of the grand-average ERP waveforms and the R^2^ plots, we chose PO7 and PO8 for the analysis of the N170 component (150–300 ms) and Fz and Cz for the analysis of the VPP (150–300 ms). The P300 was determined in the timeframe between 200 and 400 ms at electrodes POz and Oz. In addition, we analyzed the later frontal positivity in the time frame between 350 and 550 ms at Cz, and refer to this component as late positive potential (LPP) to distinguish it from the earlier parieto-occipital P300 component. We analyzed the peak amplitudes in the given timeframes and determined the peak latencies (from stimulus onset to the maximum or minimum amplitude). To compare amplitudes between display modalities repeated measures ANOVAs were conducted with condition (three levels: monitor, glasses A, glasses B) and electrode site as within-subject factors for each ERP component. If appropriate, *p*-values were corrected with Greenhouse-Geisser correction. *Post-hoc t*-tests were performed and Bonferroni corrected. Due to the longer response time of the VR headset as compared to the monitor, which is in the range between 30 and 50 ms (LaValle, 2013), we refrained from a statistical analyses of the latencies and only report the observed values.

## Results

### Performance of healthy participants

**Figure 2:**
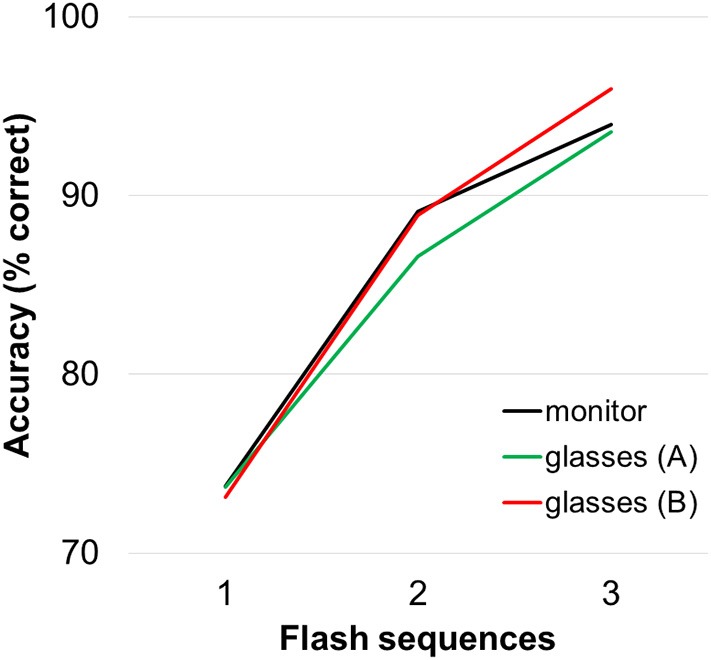
**Average accuracies for the three display methods**. Accuracies were calculated offline (for three flash sequences the values are equal to those achieved during online spelling).

Average accuracies for spelling with the three display modalities are depicted in Figure 2 for healthy participants. During online spelling with three flash sequences, participants achieved on average 94% (15.5 bits/min) correctly selected letters with both the monitor (±5.3) and the glasses A condition (±8.8%) and 96% (16.2 bits/min) in the glasses B condition (±5.8%). Data of individual participants is listed for all conditions in Supplementary Table 1. The repeated measures ANOVA revealed a main effect of “flash sequence” *F*_(1.22, 20.70)_ = 97.72 Greenhouse-Geisser corrected (GG), *p* < 0.001 but no main effect of “condition” on spelling performance *F*_(2, 34)_ = 0.11, *p* = 0.899 and no significant interaction [*F*_(1.98, 33.71)_ = 0.397, *p* = 0.673]. The *post-hoc* comparisons for number of flash sequences were all significant (*p* < 0.001).

During online spelling with one flash sequence (only performed for the glasses A condition), participants achieved on average 64% (±24.4%) correctly selected letters (32.1 bits/min). This equals 9.93 correct symbol selections per minute. Ten of the 18 participants achieved accuracies higher than 70% and the maximum bitrate (100% spelling accuracy) achieved by one participant was 71.89 bits/min.

**Figure 3:**
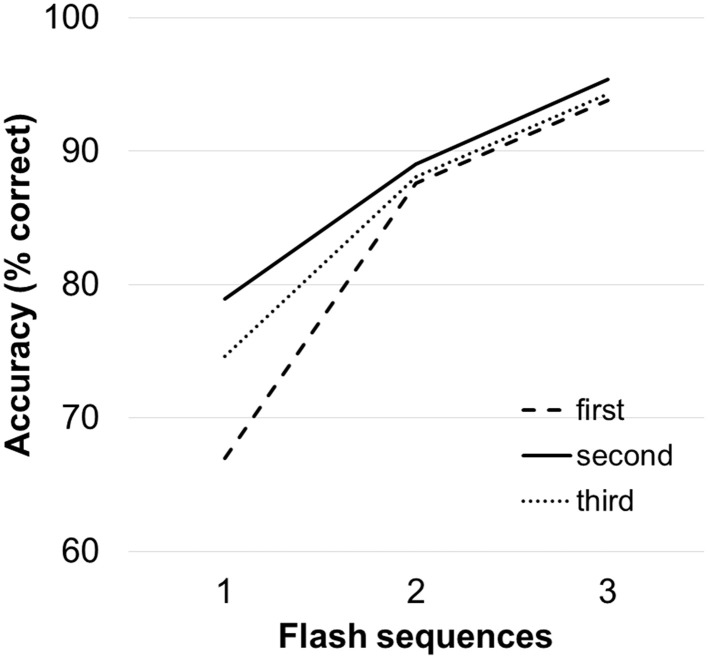
**Average accuracies as a function of order of presentation (first, second, third) independent of the display method**. Accuracies were calculated offline (for three sequences these values are equal to the accuracies achieved during online spelling).

Figure 3 depicts the average accuracies achieved by the users by order of presentation. The repeated measures ANOVA revealed a main effect of “number of flash sequences” *F*_(1.22, 20.70)_ = 97.72, *p* < 0.001 GG corrected, but no main effect of “order of presentation” on spelling accuracy *F*_(2, 34)_ = 1.44, *p* = 0.252. The “order of presentation” × “number of flash sequences” interaction was significant [*F*_(2.09, 35.67)_ = 4.16, *p* = 0.022, GG corrected). For one flash sequence, performance was higher for the condition that was presented second (79%) compared to the condition that was presented first (67%; *p* = 0.020).

### Remarks of users concerning the usability of the display methods

A total of five participants commented on the usability of the different display methods. After having first used the VR headset (glasses A) followed by the monitor, one user stated that he found it more difficult to focus on the target letter in the monitor condition and said that his eyes were “drifting away.” Nevertheless, spelling accuracy was 92% for this user in the monitor condition. Four users indicated negative aspects of using the VR headset. Two comments concerned the display quality/resolution of the headset, which one user described as “pixelated” and another noted that the letters in the margins of the matrix were “a bit out of focus.” The other two comments concerned the wearing comfort of the headset, one participant speculated that it might be “heavy if wearing it for a longer time” and one said that it was “a bit warm underneath the headset.”

### Performance of the participant in LIS

**Figure 4:**
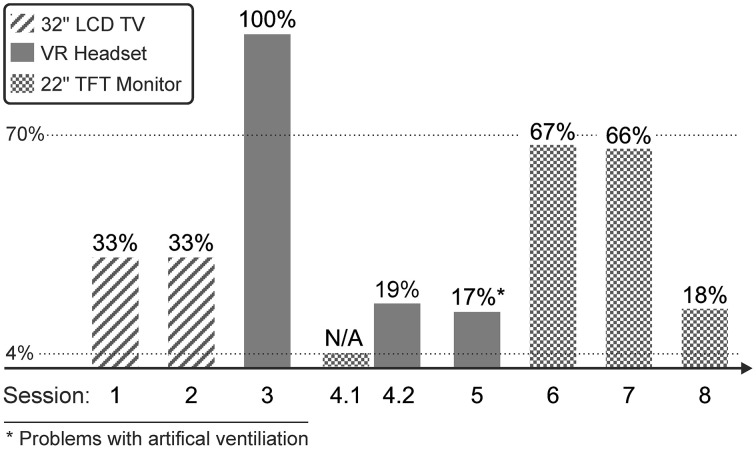
**Average accuracies achieved by the participants with LIS during online spelling in individual sessions**. Please note that the letters spelled varied between 15 and 29 for individual sessions. In session 4.1 no online spelling was performed with the 22” monitor due to the low classification accuracy achieved with the screening runs (see Section Remarks of Users Concerning the Usability of the Display Methods). The first dotted horizontal line indicates the chance level (4%) and the second horizontal line the threshold level that has been defined for satisfactory communication (70%).

The accuracies achieved during online spelling (with feedback) with different display methods in individual sessions are depicted in Figure 4 (see Table 1 for the number of letters that were spelled and details on the paradigm used). In session 4, no online spelling was performed with the 22” monitor because only 10% classification accuracy were achieved with the 10 letters acquired during the screening runs, this value only increased to 27% with additional five letters. In the same session, classification accuracy with the VR headset was 70% (for the same 10 letters that had to be copied in the screening runs with the 22” monitor) and thus, online spelling was performed with the VR headset. In session 5, problems with the artificial ventilation occurred that could have influenced the results.

### EEG data

**Figure 5:**
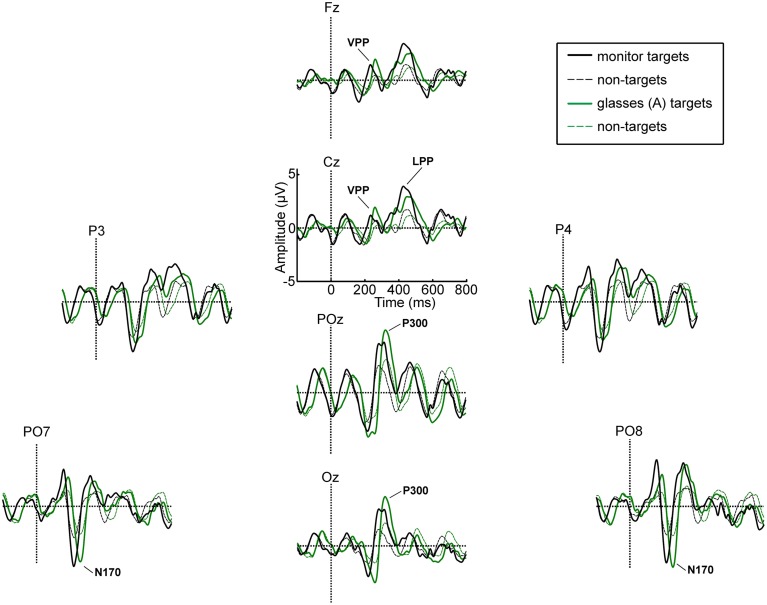
**Grand average ERP waveforms for targets and non-targets for the monitor and glasses A condition**.

**Figure 6:**
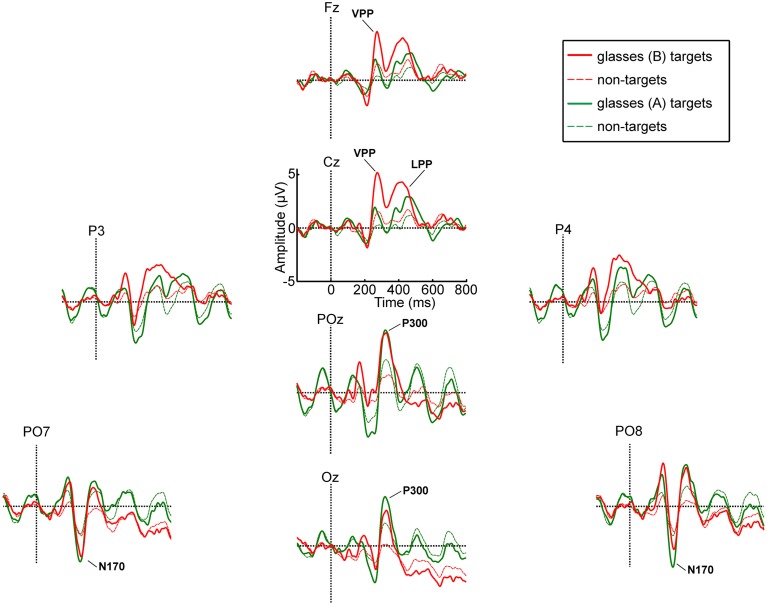
**Grand average ERP waveforms for targets and non-targets for the glasses A and glasses B condition**.

**Figure 7:**
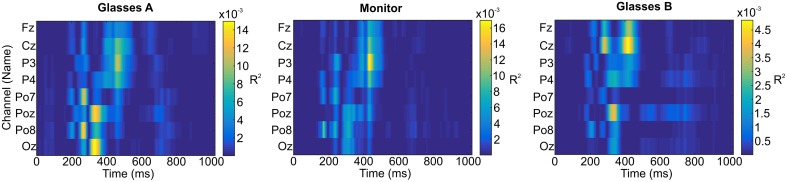
*****R***^2^ plots for the glasses A condition, the monitor and the glasses B condition**.

Figure 5 displays the ERP waveforms for healthy participants for all electrodes and the monitor and glasses A condition. Figure 6 displays the ERP waveforms for the glasses B condition and again includes the waveforms of the glasses A condition to facilitate comparison. Amplitudes and latencies for the VPP, N170, and P300 and LPP components are listed in Table 2. Figure 7 shows the *R*^2^ plots for the three conditions. It is apparent that most class discriminant information is in the time frame between 200 and 500 ms post stimulus onset independent of the condition. The distribution of the class discriminant information within this timeframe is very similar for the monitor and glasses A condition. However, the magnitude of the class discriminant information varies between the conditions for certain channels and time frames. The N170 component at PO7 and PO8 and the P300 at POz and Oz has higher discriminative values for the glasses A condition compared to the monitor. For the glasses B condition, the high magnitude of the VPP and LPP at Cz is apparent compared to the other two conditions.

**Table 2:** **Mean amplitude and latency values and standard deviations for the VPP, N170, and P300 components at selected electrode positions**.

		**VPP**		**N170**		**P300**		**LPP**
		**Fz**	**Cz**	**PO7**	**PO8**	**POz**	**Oz**	**Cz**
Monitor	*Amplitude* (μ*V*)	2.40 (±2.15)	2.70 (±2.14)	6.12 (±3.12)	5.28 (±2.92)	6.26 (±3.22)	5.24 (±3.26)	4.92 (±2.59)
	*Latency* (ms)	229.24 (±37.49)	221.68 (±38.16)	225.04 (±12.95)	221.39 (±12.29)	302.26 (±34.34)	295.89 (±41.34)	430.36 (±46.30)
Glasses A	*Amplitude* (μ*V*)	2.44 (±1.84)	2.97 (±2.08)	6.02 (±3.97)	6.18 (±4.35)	6.69 (±3.70)	5.48 (±2.62)	4.13 (±2.45)
	*Latency (ms)*	250.06 (±28.86)	240.55 (±35.73)	256.35 (±20.62)	251.29 (±23.57)	322.13 (±40.61)	315.44 (±32.79)	451.67 (±45.84)
Glasses B	*Amplitude* (μ*V*)	4.86 (±2.92)	6.16 (±3.11)	5.39 (±4.19)	4.58 (±4.82)	6.99 (±2.79)	5.09 (±2.43)	5.61 (±3.32)
	*Latency (ms)*	256.93 (±39.97)	262.39 (±32.18)	256.95 (±20.41)	247.39 (±37.87)	293.21 (±53.04)	292.57 (±51.49)	417.89 (±45.34)

*Please note that the VR headset has a delayed response time (30–50ms) that one should be aware of when comparing latencies between the display conditions*.

For the VPP, the ANOVA revealed main effects of condition [*F*_(2, 34)_ = 22.58, *p* < 0.001] and electrode site [*F*_(1, 17)_ = 11.01, *p* = 0.004] and a significant condition × electrode site interaction [*F*_(1.39, 25.51)_ = 6.11, *p* = 0.014, Greenhouse-Geisser corrected]. The VPP was significantly larger for the glasses B condition as compared to the glasses A condition (*p* < 0.001) and to the monitor (*p* < 0.001). It was significantly larger at Cz as compared to Fz (*p* = 0.004). At electrode Fz the amplitude was larger for the glasses B (4.86 μV) as compared to the glasses A condition (2.44 μV, *p* < 0.001) and to the monitor (2.40 μV, *p* < 0.001). Similarly, at electrode Cz it was larger for the glasses B condition (6.16 μV) as compared to glasses A (2.97 μV, *p* < 0.001) and the monitor (2.70 μV, *p* < 0.001).

For the N170 amplitude, the ANOVA yielded no significant results, neither a main effect of condition [*F*_(1.42, 24.19)_ = 0.99, *p* = 0.382, Greenhouse-Geisser corrected] nor of electrode site [*F*_(1, 17)_ = 1.46, *p* = 0.243] nor a significant interaction [*F*_(2, 34)_ = 1.51, *p* = 0.236].

For the P300, no significant main effect of condition [*F*_(2, 34)_ = 0.12, *p* = 0.891] was found, but a significant main effect of electrode site [*F*_(1, 17)_ = 13.28, *p* = 0.002]. The amplitude was significantly larger at POz as compared to Oz (*p* = 0.002). The interaction between condition and electrode site was not significant [*F*_(1.38; 23, 38)_ = 2.07, *p* = 0.159, Greenhouse-Geisser corrected].

The amplitude of the LPP was not significantly different for the three display modalities as revealed by the ANOVA [*F*_(2, 34)_ = 2.09, *p* = 0.139].

**Figure 8:**
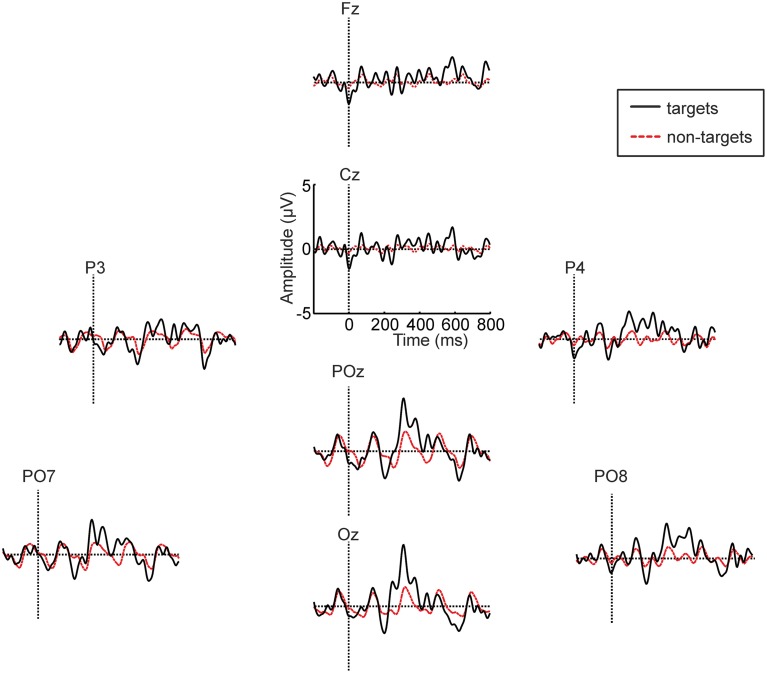
**Averaged ERP waveforms for the participant with LIS in session 3 (glasses A condition)**. The data of the copy spelling and the two free spelling runs were combined for the analysis.

Figure 8 depicts the averaged ERP waveforms for the participant with LIS in session 3. In this session she achieved 100% spelling accuracy with the VR headset (glasses A).

## Discussion

### Performance of healthy users

It could be shown that the average accuracies achieved during online spelling by the healthy participants did not differ for the VR headset and a conventional 22” TFT monitor. An accuracy of 94% (16 bits/min) was achieved with only three flash sequences during online spelling. An accuracy of 70% was previously defined as a criterion level for satisfactory BCI performance (Kübler et al., 2001). Offline classification revealed that average accuracies were above this value with only one flash sequence (single trial analysis of EEG). Thus, speed of selection could be substantially improved compared to the study by Takano et al. (2011). The authors used a P300 BCI matrix displayed with a see-through head-mounted display or an LCD monitor to control a light and a TV. They reported average online accuracies ranging from 76 to 88% with 15 flash sequences and at least 6 flash sequences were needed to reach ≥70% accuracy (offline analysis).

We could further show that online spelling with one flash sequence is feasible with the VR headset. Healthy participants achieved 64% (32.1 bits/min) in this condition during copy spelling of 18 letters. About half of the participants achieved a level needed for satisfactory spelling (>70% accuracy).

In contrast to previous studies we did not flash the characters of the matrix directly, instead rows and columns were overlaid with smileys. Kaufmann et al. (2011, 2013) showed that flashing characters with famous faces significantly improved classification performance. Here, we demonstrated that similar speed of communication and classification accuracies as reported in the studies by Kaufmann et al. can be achieved by applying simple smileys as stimulus material. About three flash sequences were needed in their study to achieve 100% classifier accuracy as compared to five for the classic flashing paradigm. A systematic comparison of monochromatic face stimuli and smileys was recently published by Chen et al. (2015). Participants of their study achieved similar accuracies and bitrates for smileys (94%, 33.3 bits/min) and human face stimuli (96%, 35.9 bits/min). Further, they did not find significant differences in face specific ERP components between the different stimuli types. This demonstrates that smileys can be an adequate stimulus to achieve state of the art ERP-BCI performance.

Most ERP-BCI studies highlight the rows or columns by flashing the characters directly (e.g., Farwell and Donchin, 1988; Sellers and Donchin, 2006; Sellers et al., 2006, 2010; Nijboer et al., 2008; Guger et al., 2009).

To our knowledge Lenhardt et al. (2008) reported the highest bitrates achieved with a non-invasive ERP speller to date using the classic flashing paradigm in which the symbols are highlighted directly. For calculation of the information transfer rate, the authors did not take the pause between letter selections into account. However, they report accurate symbol selections per minute (SPM) for both online and offline performance. Participants in their study had to copy spell 22 letters using a P300 BCI with a dynamic stopping method (number of stimulus repetitions were optimized based on different thresholds). In the best setting 4.41 letters were correctly selected per minute. In our study, 9.93 letters could be correctly selected per minute, making it a fast and competitive non-invasive ERP based BCI. Whether this rate can be maintained during sustained spelling with the BCI remains subject of further research.

The peak information transfer rate of 71.89 bits/min (100% spelling accuracy during spelling of 18 letters) achieved by one subject in the glasses A condition is in the range of invasive BCIs. In the study by Brunner et al. (2011) the intracranial EEG was recorded with Electrocorticography (ECoG,). For spelling of 43 letters an information transfer rate of 69 bits/min (86% correctly selected letters) was achieved by the participant of the study. Speier et al. (2013) reported ITRs of 49.47 bits/min and 27.05 bits/min respectively, achieved by the two epilepsy patients that participated in their study.

Although speed of communication with an ERP BCI can already be quite high using salient stimulus material, it can be optimized further. In a proof-of-principle study, Kaufmann and Kübler (2014) demonstrated that it is possible to double the spelling speed by modifying the paradigm. Two very distinct types of stimuli were superimposed on different characters simultaneously during flashing. The offline analysis revealed that about half of the participants could improve their performance compared to a single stimulus (face) paradigm and two users could achieve information transfer rates of 150 bits/min.

#### Performance in the glasses B (single stimulus) condition

Participants achieved high accuracies (96%) during online spelling with the glasses B condition. In this condition, only the target letters were in the field of view of the user. Therefore, it constitutes a “single stimulus paradigm” (Polich et al., 1994). Although this display method did not yield higher accuracies as compared to the other two “oddball conditions” (glasses A and monitor), probably due to a ceiling effect, it might be an alternative display option for healthy users. In this condition, individual stimuli are particularly large and hard to ignore and therefore less mental effort is required for their recognition. This configuration of the VR headset cannot be used by persons with severe paralysis and unable to move their heads. However, for users with severe paralysis, but able to move their heads, the possibility to infer the head position with the VR headset could be advantageous. For instance, it could be used to display different matrices depending on the direction of view of the user and thus, speed of communication could be improved. Additionally, BCI controlled applications could be displayed in addition to the BCI matrix in different locations of the user's field of view that currently need two screens, such as the web browser proposed by Halder et al. (2015).

### Performance of the participant in LIS

Online spelling accuracy varied substantially for different display methods and sessions. There are many factors that can negatively influence BCI performance, particularly during home use by a participant with LIS: e.g., artificial ventilation, fluctuations in health condition, noisy and distracting environment, side effects of medication. Nevertheless, the participant was able to spell 15 letters with an online accuracy of 100% during one session, demonstrating that an 80 year old person in the locked-in state is able to use the VR headset to control an ERP BCI. In another session, a classifier performance of 70% was achieved with the VR headset, although in the same session classifier accuracy with a conventional monitor was only 10%. Taking into account all sessions and accuracies achieved with the different display methods, one can state that similar accuracies can be achieved with the VR headset as compared to conventional display methods (large screen TV, flatscreen monitor). Thus, we propose the VR headset as a display option for situations in which mounting a conventional monitor might be difficult or not feasible. Nevertheless, the tested VR headset has several disadvantages, which are discussed in the next section.

### Usability of the different display methods

Unlike a conventional monitor, the VR headset (Glasses A condition) has the advantage of always displaying the speller matrix in the field of view of the user. A disadvantage is that communication is no longer possible via eye movements once the user is wearing the VR headset. We made sure that the participant felt comfortable wearing the headset by lifting it after every word spelled. If using the headset for a longer period of time, it would visually isolate the user from his or her environment. Further, healthy participants pointed out that it might be uncomfortable to wear for longer periods of time due to its weight and because it might get warm underneath the headset. An additional disadvantage is that the resolution of the VR headset is low. Therefore, the tested version of the VR headset might be particularly suited for initial communication attempts with a BCI for severely paralyzed persons if mounting a monitor is difficult. For long term use of a P300 BCI, displaying stimuli on a conventional monitor is probably advantageous for social-interactive reasons.

Usability of head-mounted displays will likely be improved in the near future (e.g., reduced weight, higher screen resolution, high quality see-through displays) and therefore become an even more promising display option for BCI use. Further, the combination of a BCI and a virtual environment presented via a HMD (as explored by Leeb et al., 2006) might be of particular interest for persons who are severely paralyzed to gain a higher quality of life.

### ERP waveforms

In the present study, we did not systematically investigate the effects of the stimulus material (smileys) on ERP waveforms. The lack of a control conditions such as flashing rows and columns with the face stimuli proposed by Kaufmann et al. (2011, 2013) or highlighting the rows and columns directly, does not allow us to draw definite conclusions on the effects of stimulus material on ERP waveforms. However, we investigated whether the face specific ERPs reported by Kaufmann et al. (2011, 2013), namely the N170 and the N400f were also apparent in our study using smileys as stimulus material.

In the grand-average ERP waveforms, the most pronounced negative deflection is apparent at electrode positions PO7 and PO8 for all display methods. Although the mean latencies for the monitor condition at PO7 (225 ms) and PO8 (221) are substantially later (~50 ms) than the values usually reported in studies of face perception (e.g., Bentin et al., 1996; Eimer, 2000), we argue that they are not atypical and this negativity is the N170 component. First of all, even longer latencies were observed for a face specific negative component by Luo et al. (2010) in a rapid serial visual presentation (RSVP) task in response to faces with different emotional expressions (240 ms) and by Chen et al. (2015), who compared the ERPs elicited by faces and smileys using a P300 based BCI (252 ms). Secondly, in the same timeframe as the N170 component, a vertex positive potential (VPP) was apparent at electrode positions Cz and Fz in our study. Thus, the N170 at parieto-occipital electrode sites and the VPP at fronto-central electrode sites were apparent at the expected sites. As stated in the introduction, Joyce and Rossion (2005) provided strong evidence that they are manifestations of the same neural generator. No clear N400f component was apparent at central and parietal electrode sites in our study. Since the component is linked to processes involved in the recognition of familiar faces, this potential was not expected (Eimer, 2000). Hence, we focused on the analysis of the N170 and VPP components.

The ANOVA did not reveal a significant difference between the amplitudes of the N170 between the three display modalities, but the amplitude of the VPP was significantly larger for the glasses B condition as compared to the other two conditions. The glasses B condition differed from the other two conditions twofold: firstly, only the targets were seen (single stimulus, rather than conventional target/non-target oddball condition) and secondly, individual stimuli filled the field of view of the user, thus, stimulus size was substantially larger. Hence, it is easier to focus on the targets and they are more salient. To our knowledge, no studies reported on manipulating the size or discriminability of smileys/stimuli in a P300 BCI task, but several basic studies point in the direction that the positive component in the timeframe of the VPP (150–250 ms) can be manipulated by stimulus properties, with higher stimulus intensity eliciting a higher P200 (Picton et al., 1974; Vesco et al., 1993; Sugg and Polich, 1995; Covington and Polich, 1996). The majority of these studies manipulated stimulus intensity in the auditory domain.

The P300 was largest for the glasses B condition, but unlike hypothesized, it was not significantly larger as compared to the other two conditions. Whether larger stimuli in a visual “single stimulus” paradigm elicit larger P300 amplitudes has not yet been systematically investigated. Li et al. (2011, 2014) investigated the effect of screen size and stimulus luminosity with a P300 BCI and found that a computer monitor elicited a larger P300 as compared to a cell phone display and reported a higher P300 with increased luminosity of the stimuli, the latter effect, however, was not significant. Similar P300 amplitudes were elicited by a visual oddball and a visual “single stimulus” paradigm (Mertens and Polich, 1997).

We conclude that a systematic study is needed to investigate the effects of using a “single stimulus” paradigm and determining the effect of stimulus size on ERP waveforms with a P300 BCI.

### General remarks

We found a significant interaction of “order of presentation” and “number of flash sequences.” This demonstrates that the order of presentation (of different display methods) can have an influence on spelling accuracy with a BCI, particularly for few stimulus repetitions. Therefore, it is crucial to control for order effects, when comparing different experimental conditions. Because we controlled for order effects and did not find a main effect of display method, the finding suggests that participants performed particularly well with the BCI after they have gained control over it (after the first condition) independent of display method.

## Conclusions

Healthy users achieved very high spelling accuracies (>90%) with a VR headset (head-mounted display), similar to those achieved with a conventional monitor used to display a P300 BCI matrix. A person in the locked in state was able to gain control over the BCI (100% in one session) using the VR headset. Therefore, we propose it as a display option for severely paralyzed persons for situation in which mounting a conventional monitor is not feasible.

We also demonstrated that rapid BCI communication is possible with only one flash sequence (single trial analysis of EEG) using the VR headset in some but not all subjects. With 9.91 correctly spelled letters per minute it constitutes a fast and competitive ERP BCI.

### Conflict of interest statement

The authors declare that the research was conducted in the absence of any commercial or financial relationships that could be construed as a potential conflict of interest.

## Supplementary material

Table S1 **Performance for each participant and for all conditions listed by the number of flash sequences**. Online spelling with one flash sequence was only tested for the glasses A condition.
